# The exploratory value of cross-sectional partial correlation networks: Predicting relationships between change trajectories in borderline personality disorder

**DOI:** 10.1371/journal.pone.0254496

**Published:** 2021-07-30

**Authors:** Lino von Klipstein, Denny Borsboom, Arnoud Arntz

**Affiliations:** 1 Department of Psychiatry, Interdisciplinary Center Psychopathology and Emotion Regulation (ICPE), University of Groningen, University Medical Center Groningen, Groningen, The Netherlands; 2 Department of Psychological Methods, University of Amsterdam, Amsterdam, The Netherlands; 3 Department of Clinical Psychology, University of Amsterdam, Amsterdam, The Netherlands; University of Pennsylvania Perelman School of Medicine, UNITED STATES

## Abstract

**Objective:**

Within the network approach to psychopathology, cross-sectional partial correlation networks have frequently been used to estimate relationships between symptoms. The resulting relationships have been used to generate hypotheses about causal links between symptoms. In order to justify such exploratory use of partial correlation networks, one needs to assume that the between-subjects relationships in the network approximate systematic within-subjects relationships, which are in turn the results of some within-subjects causal mechanism. If this assumption holds, relationships in the network should be mirrored by relationships between symptom changes; if links in networks approximate systematic within-subject relationships, change in a symptom should relate to change in connected symptoms.

**Method:**

To investigate this implication, we combined longitudinal data on the Borderline Personality Disorder Severity Index from four samples of borderline personality disorder patients (*N* = 683). We related parameters from baseline partial correlation networks of symptoms to relationships between change trajectories of these symptoms.

**Results:**

Across multiple levels of analysis, our results showed that parameters from baseline partial correlation networks are strongly predictive of relationships between change trajectories.

**Conclusions:**

By confirming its implication, our results support the idea that cross-sectional partial correlation networks hold a relevant amount of information about systematic within-subjects relationships and thereby have exploratory value to generate hypotheses about the causal dynamics between symptoms.

## Introduction

The network approach to psychopathology challenges the traditional conceptualization of pathologies as latent mental disorders (e.g., depression) that are the common cause to the occurrence of a group of symptoms (e.g., sleeping problems, fatigue). Instead, this approach conceptualizes pathology as complex systems of symptoms with direct causal and homeostatic links between each other (e.g., sleeping problems cause fatigue) [1, 2]. Mental disorders are not understood as latent variables that can be measured through the symptoms they cause, but as groups of symptoms that show strong relations among each other so that they form a cluster. Thereby, symptoms do not *measure* mental disorders but *constitute* mental disorders. With these ideas, the network approach has brought us an etiological and maintenance model of psychopathology with a new focus–single symptoms and the relationships between them.

In the past years, a number of methods have been developed that estimate the relationships between symptoms [3–5]. The most prominent method has been cross-sectional partial correlation networks (CPCNs), which estimate partial associations between symptom-pairs controlling for all other symptoms in the network. CPCNs can be estimated with high specificity and efficiency through Markov Random Fields [6]. In the estimation, spurious associations (i.e., those due to sampling variation) are suppressed through regularization [7]. Graphical representations of such networks–where symptoms are represented as *nodes* and relations between symptoms as *edges* between nodes–provide an interpretable visualization of the data structure. Ultimately, CPCNs estimate a conditional (in-) dependence structure, that is theorized to be indicative of the causal structure that underlies it [1].

When looking at estimated CPCN, researchers have shown interest in the role that single symptoms play in the larger network. That is, in a (theoretically) causal symptom network each single symptom has unique causal relations to other symptoms. Therefore, different symptoms play different roles–some more important than others. One may conceptualize importance in this context as the extent to which the activity of a symptom affects the activity in the overall network (i.e., the larger pathology). Proponents of the network approach have proposed that so-called centrality parameters can indicate this importance of symptoms [1]. Centrality parameters describe different aspects of how single symptoms in the estimated network are connected to other symptoms. The most commonly applied parameters are *strength*, *betweenness*, and *closeness*.

In summary, advocates of the network approach have claimed that CPCNs can approximate the pattern of causal relationships between symptoms, which can, in turn, be used to identify important symptoms using centrality parameters. These claims are independent of the theoretical value of the network approach as a theory of psychopathology, but central to its application in research. The network approach relies on CPCNs as a frequently applied network analysis [8–10]. Further, identifying important symptoms through centrality parameters has been a central argument for the clinical utility of network analyses, as it may suggest interesting treatment targets [8, 10, 11].

There are good reasons to doubt that CPCNs can deliver on these claims. Firstly, CPCNs are purely correlational and can thereby never by themselves serve as evidence of causal relationships. In response to this, one may defend CPCNs by invoking that nobody really claims that the edges found in CPCNs are a direct representation of causal processes. Instead, researchers have often referred to the *exploratory value* of CPCNs [7, 12]. That is, edges in networks can be used to generate hypotheses about causal relationships but not more. However, even in such an exploratory framework, CPCNs may not live up to the claims described above.

The reason for this is that CPCNs are between-subjects analyses. As Molenaar and others argued, the structures of between-subject variation and within-subject variation are only equivalent under very stringent conditions, which are utterly unrealistic in psychological research [13–15]. This makes inferences based on relations found in between-subject analyses (like edges in CPCNs) about within-subject processes (like direct causal effects between symptoms) problematic. This problem has also received attention in the network literature [16, 17] and was often paired with a call for methods that estimate within-subject relationships from individual time series. Following this argument, edges in CPCNs can clearly not serve as direct representations of within-subject relationships (causal or otherwise). However, such direct representation may be an unnecessarily strict criterion within an exploratory framework. That is–as any between-subject analysis generally contains both between-subject and within-subject variation [18]–if CPCNs represent within-subject variation *to a significant degree* and thereby serve as an approximation, they hold exploratory value.

Let us assume that CPCNs indeed (to a significant degree) represent systematic within-subject relationships. With “systematic” we mean non-spurious relationships, that are the result of some within-subject causal mechanism (e.g., direct links, like network theory proposes, indirect links, or a common cause). Under this assumption, change in symptoms that are related in the network should be related as well. More specifically, if edges in a between-subject CPCN approximate within-subject relationships, change in a symptom should be most strongly related to change in the symptoms that are most strongly connected to it in the network. Further, if centrality parameters can really identify important symptoms, change in a high-centrality symptom should be more strongly related to change in the remaining overall pathology (i.e., the aggregate severity of all other symptoms) than change in a low-centrality symptom. Note that we do not conjecture that the implication, that more central symptoms turn out more predictive, would in itself be strong evidence for network theory; various other models (e.g., factor models) are likely to imply similar patterns of dependency, and correlational data is rarely strong enough to distinguish these candidate explanations [6, 19]. It would, however, support a systematic relation between longitudinal change patterns and cross-sectional patterns of association that is of interest in itself and that might be exploited in applications.

The present study explored whether a CPCN can predict such change relations in a large dataset compiled of individuals with borderline personality disorder (BPD), who provided longitudinal measurements on their symptoms. In a first step, we used baseline measurements of symptoms to estimated CPCNs and centrality parameters. In a second step, we investigated how the change trajectory in single symptoms is related to (a) change trajectories in other symptoms and (b) the change trajectory in the remaining overall BPD pathology. For this purpose, we looked at relationships between random slope parameters (i.e., estimates of change trajectories) estimated in multivariate multilevel linear regression models. In a third and final step, we related network parameters from the first step to the relationships between change trajectories from the second step.

## Method

### Sample

The present study used a sample comprised of existing datasets providing longitudinal measurements on the Borderline Personality Disorder Severity Index, 4th version (BPDSI-IV) [20]. The only other criteria for including data were ethical approval and authors’ consent. Data from four sources was included, all of them treatment trials targeting BPD: 18 subjects stem from a study by Dickhaut and Arntz [21], 86 subjects from a study by Giesen-Bloo and colleagues [22], 71 subjects from the Oulu BPD Study [23], and 508 subjects from data collected before 12^th^ May 2016 in the ongoing trial by Wetzelaer and colleagues [24]. All of these samples consisted solely of BPD patients undergoing treatment. In the four studies patients received different treatments and followed different measurement schedules: one study measuring every 6 months for 2.5 years in patients receiving a combination of individual and group schema therapy [21]; one study measuring ever 3 months for 3 years in patients receiving either schema therapy or transference-focused psychotherapy [22], one study measuring twice (at 0 and 12 months) in patients receiving either community treatment by experts or treatment as usual [23], and one study measuring at 0, 6, 12, 18, 24, and 36 months in patient receiving group schema therapy, a combination of individual and group schema therapy, or community treatment by experts [24]. For detailed inclusion and exclusion criteria, please refer to the original publications.

### Measure

The BPDSI-IV is a semi-structured interview comprised of 70 items that are based on the nine BPD symptoms defined in the DSM-IV. A subscale can be calculated for each DSM-IV symptom. Interviewers rate the frequency of symptoms on an 11-point scale from “never” to “daily”. The BPDSI-IV has shown good reliability and validity [20, 25]. Interviews were conducted by trained research assistants [21, 22, 24] or health care professionals [23].

### Statistical analysis

All analyses were run both on the items of the BPDSI-IV and its nine subscales, representing the DSM-5 BPD criteria. To simplify, we will use *BPDSI elements* to refer to both items and subscales in the following description of the analyses.

#### Baseline network estimation

CPCNs of BPDSI elements at baseline were estimated as Markov Random Fields, where edges between nodes represent partial associations controlling for all other items in the network. More specifically, we applied Gaussian Random Fields, assuming normality of symptom frequency. Pearson correlations after nonparanormal transformation [26] provided the input for the estimation of networks. A regularization technique was employed to retrieve networks without connections that are likely spurious (i.e., due to sampling variation). To this end, network model parameters were estimated using the Least Absolute Shrinkage and Selection Operator (LASSO) [27] with the Extended Bayesian Information Criterion [28] for model selection. For an accessible introduction to the estimation of regularized CPCNs see Epskamp and Fried [7]. On the basis of the estimated networks, centrality parameters were calculated. Network analyses were conducted using the R-package qgraph [29]. Graphical representations of the network were created using the Fruchterman-Reingold algorithm [30]. R-code for all steps in the analysis can be found in the (S1 Data).

#### Relating change to change

Change in BPDSI elements was modeled using multivariate multilevel linear regressions. We chose multilevel regressions because our data is hierarchical. That is, measurements are clustered within individuals, who are clustered within studies. Within such clusters the assumption of independent observations is violated. To account for this, multilevel models include the hierarchical structure by distinguishing between fixed and random effects. That is, they estimate overall regression parameters across clusters (fixed effects) while including residual terms that describe the deviation from these fixed parameters due to clustering (random effects). For example, for multiple individuals with longitudinal measurements (measurements clustered within individuals) fixed intercept and slope parameters describe the average development over time in the overall sample, while random effects describe the deviation of each individual’s slope and intercept from those fixed parameters. Multilevel regression models allow for the analysis of data with longitudinal measurement occasions varying in number and spacing [31]. This makes them especially advantageous for the analysis of data taken from different studies that use different measurement protocols. We chose to apply a multivariate version of multilevel linear regression because this allowed us to directly estimate the correlation between individual change trajectories (random slopes) in a pair of variables. For an introduction to multivariate multilevel linear regression for clinical researchers see Baldwin, Imel, and Braithwaite [32].

Separate models were estimated for each BPDSI element. Each model incorporated two dependent variables: the relevant BPDSI element and a rest-score (total BPDSI score excluding the relevant BPDSI element). Models had a 4 level structure, where level 1 modeled the relevant dependent variable using dummy variables, level 2 modeled intra-individual change over time, level 3 modeled inter-individual differences, and level 4 modeled differences between studies. The models estimated fixed and random effects in intercepts and slopes for individuals and studies. This results in the following model equation:

$$Y_{hijk}=[\gamma_{1000}+v_{100k}+u_{10jk}+(\gamma_{1111}+v_{111k}+u_{11jk}){time}_{1ijk}+e_{1ijk}]d_{1ijk}+[\gamma_{2000}+v_{200k}+u_{20jk}+(\gamma_{2111}+v_{211k}+u_{21jk}){time}_{2ijk}+e_{2ijk}]d_{2ijk}$$ (1)

*Y*_*hijk*_ refers to the response on dependent variable *h*, at time point *i*, in individual *j*, in study *k*; *d*_*1ijk*_ and *d*_*2ijk*_ are dummy variables indicating the relevant dependent variable; *γ*_*1000*_ and *γ*_*2000*_ represent the average values of the respective dependent variable at baseline; *γ*_*1111*_ and *γ*_*2111*_ represent the average change in the respective dependent variable per month; *v* represents random intercepts and slopes varying between studies; *u* represent random intercepts and slopes varying between individuals; and *e* represents the residuals. The models estimated all elements in the variance-covariance matrices of random effects and residuals.

We applied Bayesian statistics and Markov chain Monte Carlo (MCMC) methods to estimate the model parameters, using the R-package MCMCglmm [33]. We chose inverse-Wishart priors as the prior distributions [34]. As Bayesian statistics create distributions of estimated parameters (i.e., so-called posterior distributions), we calculated the means of these distributions to acquire point estimates of the parameters.

The above models taken together estimate random slopes for each individual on each BPDSI element. We used these estimates as data to estimate random slope networks. That is, we applied the same network analyses to the estimated random slopes that we applied to baseline BPDSI data (though without nonparanormal transformation).

The above models directly estimated the relationship between change trajectories of BPDSI elements and change trajectories of the remaining BPD pathology by estimating the correlation between random slopes across individuals on the two dependent variables, cor(*u*_*11jk*_, *u*_*21jk*_).

#### Relating parameters from baseline network to change relationships

Ultimately, we related network parameters from baseline CPCNs to change relationships from the multivariate multilevel linear regression models on two levels: Firstly, we related the baseline CPCN to the random slope network by calculating Pearson correlations between their adjacency matrices (matrices containing edge weights). Secondly, we calculated Pearson correlations between centrality parameters from baseline CPCNs with the random-slope-correlations cor(*u*_*11jk*_, *u*_*21jk*_). To illustrate, one may represent this correlation as:

cor(centralityparameter,cor(randomelementslope,randomrestscoreslope)) (2)

## Results

### Sample characteristics

The combined data from the included sources amounted to a total sample of *N* = 683 individuals. The sample consisted completely of adult BPD patients and was largely female (*N* = 597; 87.4%). The average age was *M* = 32.25 (*SD* = 9.13; *range* = 18–61). The mean BPDSI score was *M* = 31.05 (*SD* = 8.73). The acquired datasets implemented different measurement schedules and all of them had some dropout. See S1 Table for the available data per measurement point for each dataset. Further, the publications on the data sources [21–23] provide additional information on dropout in their samples. Some data from Wetzelaer and colleagues [24] was incomplete because the trial is ongoing and some participants have simply not finished all measurements. Such missing data was not treated as missing but simply omitted. See S2 Table for missing data per time point due to actual dropout.

### Baseline partial correlation networks

**Fig 1:**
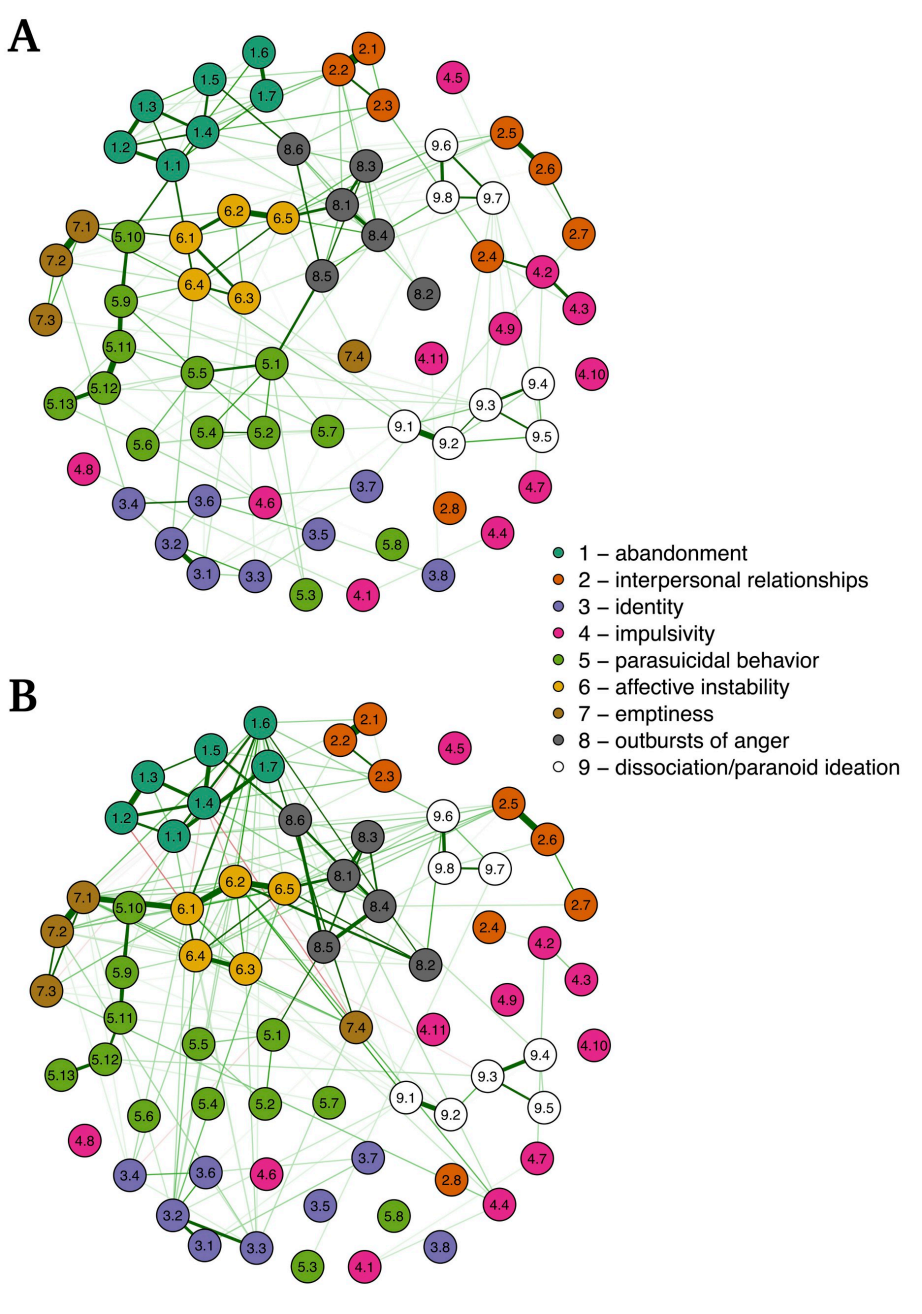
Partial correlation networks for the 70 BPDSI items. Panel A shows the network of baseline scores. Panel B shows the network of random slopes. Note that nodes in panel B are placed in the same location as in panel A for ease of comparison. Nodes are labeled in the following way: (number of subscale. item number).

In the interest of brevity, we only present networks of BPDSI items here. See Fig 1 panel A for the baseline CPCN. Networks of BPDSI subscales, as well as centrality parameters, can be found in the (S1–S4 Figs).

### Multivariate multilevel linear regression models

The multivariate multilevel linear regression models were estimated on the basis of the same sample as the baseline CPCNs and included considerable missings due to dropout. We included these missings in our analysis. Under the assumption that missing data was missing at random, the estimated models provide unbiased estimates [35]. After inspection of autocorrelations between iterations of the MCMC algorithm, we chose a thinning interval of 50. With a burn-in of 5000 and 105000 iterations, this resulted in a sample of 2000 draws in the posterior distributions of estimated parameters. Visual inspection of trace plots and histograms of posterior distributions indicated good convergence. Fixed linear time slopes are provided in S7 and S8 Figs. As we estimated 79 models with 27 posterior parameter distributions each, we refrain from presenting further results here in the interest of brevity (these results can be provided upon request). The correlations between slopes in BPDSI elements and slopes in restscores can be found in S5 and S6 Figs.

### Relating baseline network and random slope network

Fig 1 shows the baseline CPCN and the random slope network for BPDSI items side by side. The adjacency matrices of the baseline CPCN and the random slope network correlated with *r* = .83 for BPDSI items and with *r* = .74 for BPDSI subscales. These results are based on networks that underwent regularization using LASSO. To investigate the robustness of these findings, we conducted two alternative analyses. Firstly, LASSO sets many edges to zero and thereby creates a distribution of edges that is distinctly non-normal. This in turn may have affected the Pearson correlation estimates. To address this, we estimated non-regularized networks (for both slope and baseline networks) and again correlated adjacency matrices. This resulted in *r* = .64 for BPDSI items and *r* = .78 for BPDSI subscales. Secondly, we estimated networks using Bayesian methods for estimation and model selection [36], implemented in the R package BGGM [37]. This resulted in correlations between adjacency matrices of *r* = .69 for BPDSI items and *r* = .76 for BPDSI subscales.

### Relating centrality and random slope correlations

Table 1 shows Pearson correlations between (a) correlations between random slopes in BPDSI elements (i.e., BPDSI subscales and items) and random slopes in BPDSI rest-scores, and (b) three types of centrality parameters (i.e., closeness, strength, and betweenness). The results show high to medium correlations on the level of BPDSI subscales, but low correlations on the level of BPDSI items. Considering the size of the BPDSI item network, one may find it unlikely that the effect of a single element can be traced in the rest-score; even if one assumes the network truly captures causal processes. Further, one should take considerable sampling error into account. To address this post-hoc, we repeated the analysis for BPDSI items with neighborhood-scores in place of rest-scores. Neighborhood-scores were defined as the sum-score of all BPDSI items directly connected to a given BPDSI item in the CPCN. This resulted in correlations between random slope correlations and centrality parameters of *r* = .72 for closeness, *r* = .79 for strength, and *r* = .41 for betweenness (since the items 2.8 and 4.10 were not connected to other symptoms, these results are based on the remaining 68 items). To check for the potential influence of floor or ceiling effects on centrality parameters we calculated Spearman’s rank correlations between node strength and baseline standard deviations, resulting in *r* = .29 for BPDSI items and *r* = -.25 for BPDSI subscales.

**Table 1:** Correlations between centrality parameters and random slope correlations by BPDSI level.

BPDSI level	Closeness	Strength	Betweenness
subscales	.73	.55	.58
items	.39	.37	.26

## Discussion

The present study aimed to investigate the claim that CPCNs have exploratory value for generating hypotheses about the causal within-subject dynamics in psychopathology. In a sample of patients with borderline personality disorder (BPD), we examined whether association patterns found in baseline CPCNs translate to association patterns in pathology change. To this end, we firstly compared baseline CPCNs to CPCNs calculated from random slopes. Secondly, we related centrality parameters from the baseline CPCN to correlations between random slopes in single symptoms and random slopes in rest-scores.

The first part of our results showed that edges in baseline CPCNs were highly predictive of edges in CPCNs estimated from random slopes (for both levels of analysis: BPDSI items and BPDSI subscales). The second part of our results showed that centrality parameters from our baseline CPCN were strongly related to random slope correlations on the level of BPDSI subscales, but only weakly related to random slope correlations on the level of BPDSI items. However, when we repeated our analysis on the item level using neighborhood-scores in place of rest-scores, we also found strong relationships. Notably, the size of the relationships varied between centrality parameters, with closeness consistently showing high associations. Overall, parameters of the baseline CPCN were strongly predictive of association patterns in change trajectories.

If one holds the theory that CPCNs approximate systematic within-subject relationships, the pattern of results we found is a necessary implication. That is, change in high-centrality symptoms should relate to change in the remaining overall pathology and change in pairs of symptoms that are related in the network should be related. Our results confirm these implications, thereby lending some support to the theory that CPCNs approximate systematic within-subject relationships. However, note that the implication is not specific to network models and may be derived from other models as well. For example, if the data were generated by a latent variable model, the same pattern may arise if the latent variable model is invariant over time and fluctuations in scores are due to random error, and a host of other models may carry the same implication. Hence, the results should not be overinterpreted. The data show *only* that the (partial) correlation structure of within-person changes in scores is similar to the (partial) correlation structure of scores as computed on the individual differences. However, while acknowledging this, we would still argue that our results support the claim that between-subject baseline CPCNs carry a relevant amount of information about within-subject processes. Given Molenaar’s [14] argument that inferences from between-subject analyses to within-subject processes are inherently problematic, there is good reason to doubt this claim, and researchers have indeed questioned whether cross-sectional network models and related data analytic tools can have any relevance at all in describing change [16, 38, 39]. To address this issue directly in the network context, one would have to show a match between between-subject relationships and within-subject relationships, ideally using experimental interventions that directly probe the network model. Our analysis did not do that. Instead, we compared between-subject relationships with between-subject relationships in within-subject change (i.e., between-subject relations of individual/random slopes). Just as any between-subject analysis, our analysis of between-subject relations in within-subject change includes both between-subject and within-subject sources of variance [18]. However, we think it is reasonable to hypothesize that differences in individual change are largely driven by within-subject processes. To the extent that this hypothesis is true–which is, of course, debatable–our results show a match between between-subject relations and within-subject relations. In this way, while the finding does not in itself lend support to the network theory in particular, it does support the more modest claim that baseline CPCNs have some exploratory value for investigating within-subject dynamics in psychopathology and thus serves to counter the extreme position that within-subject change and between-person differences are completely unrelated. Future research may try to more directly compare between-subject relations from CPCNs with within-subject relations, which can be derived from person-specific network models [3] based on experience sampling data.

While our results point towards a substantial match of between-subject relationships and within-subject relationships, they do not in any way speak to the causal nature of these relationships. That is, these relationships could be the result of common causes, indirect causal links, latent variables, reciprocal interactions, or direct causal links. Thereby, although our results inform network methodology, as they support the exploratory use of CPCNs, they do not weigh in on network theory, which holds that the relationships are the result of direct causal links.

Independent of whether the match between our CPCNs and change relations was due to within-subject or between-subject variation, our findings speak to the predictive power of networks. Regarding centrality, Robinaugh and colleagues [10] applied an analysis similar to ours in a complicated grief network and showed that change in high-centrality symptoms is related to change in the remaining overall pathology. Our results expand on this finding by replicating it in a different pathology, while using a more elaborate statistical model (i.e., regression models instead of pre-post difference scores). Note that, besides the predictive power of centrality parameters, there are legitimate conceptual questions about what they capture [40]. Beyond the topic of centrality, the present study is the first to show a prominent match between relationships in CPCNs and relationships between long-term change trajectories. In our efforts to show this, we applied an innovative analysis approach in that we used change trajectories (random slopes) as input for a CPCN. Other researchers have demonstrated the predictive power of networks in their work. Boschloo, van Burkulo, Borsboom, and Schoevers [41] found that symptoms that were predictive of depression onset had high centrality in their baseline network. And, although the evidence is not univocal [42], van Borkulo and colleagues [43] suggest that the baseline network of participants with persistent depression was more densely connected than that of recovered participants. Further, research on critical slowing down has provided findings suggesting that longitudinal auto-correlations may serve as an early warning sign for transitions in depression [44, 45]. Taken together, this research builds a plausible case for the relevance of network characteristics, both on the zoomed in level of single symptoms and the zoomed out level of the full network.

Since we are arguing that the CPCNs we estimated hold exploratory value, it would be amiss to not at least look at some notable suggestions and hypotheses, that arise regarding BPD (though a detailed look is beyond the scope of this paper). Firstly, in the item network (Fig 1A), the edges between items of the same subscale are notable. Items in the subscales *affective instability*, *emptiness*, (fear of) *abandonment*, *outbursts of anger*, and *identity* (disturbance) overall exhibited stronger edges to other items of their subscale than to items of other subscales, suggesting that these subscales (or symptoms) largely behave as uniform constructs within the BPD pathology. Three of the BPDSI subscales (and equally their corresponding DSM criteria) each cover two aspects–*interpersonal relationships* covers partner relationships (items 2.1–2.4) and other relationships (items 2.5–2.8), *parasuicidal behavior* covers automutilation (items 5.1–5.8) and suicidal plans/attempts (5.9–5.13), and *dissociation/paranoid ideation* covers the two aspects in its name (items 9.1–9.5 and 9.6–9.8, respectively). For each of these subscales its two aspects show up separately in the item network. In *interpersonal relationships* and *dissociation/paranoid ideation* the respective aspects even show little to no direct relationships with each other. This result stands in contrast to studies supporting the nine-factor structure of the BPDSI [25] and suggests that we might be better off regarding the different aspects as separate constructs with different roles and relationships within the BPD pathology. Items of the *impulsivity* subscale show little relationships with each other and with other items, suggesting that they did not act as a uniform construct and indicating either measurement or conceptual issues with this subscale. Secondly, our results indicate that *affective instability* plays a central role within the BPD pathology as it exhibited both high centrality in networks and high relationships with the trajectory of the overall pathology on both item- and subscale-level. The latter result indicates that changes in affective instability might be an important driver (or indicator) of change in BPD during psychological treatment. This result is in line with previous network analyses on BPD [46, 47], which also showed high centrality for affective instability.

An important limitation to consider in the interpretation of the results is the time scale of the longitudinal data used in the analyses. Change trajectories over time were estimated over the course of 1–3 years with measurement intervals of 3–12 months. Interpretation is thus limited to this time scale. Further, we only provide a basic description of our sample, a limitation that should be considered for the clinical perspective we provide.

CPCNs of psychopathology symptoms can only be a first step in shaping models of psychopathology that capture the dynamics between elements of the pathology. We cannot draw conclusions about the nature or direction of relationships in CPCNs. For example, a more clinically-oriented look at our analysis suggests that affective instability plays an important role within BPD. However, we cannot say whether this result reflects that affective instability affects other symptoms, that it is affected by them, or that a third variable affects both. Even prominent and effective treatment approaches for BPD would disagree on this. While from the view of Schema Therapy (ST) affective instability is seen as the common result of other causal elements [48], Dialectical Behavior Therapy (DBT) puts dysfunctional affect regulation at the center of its theory and treatment [49]. It is thus important that researchers follow up on the implications taken from network analyses and investigate the nature of these relations. Further, most researchers have constructed their network only from symptoms, thereby disregarding other elements and a wealth of clinical theory and knowledge. To capture the building blocks of causal dynamics, networks need to be expanded. Fried and colleagues [8] provided an example of this by showing the importance of non-symptom elements in a depression network. In the case of BPD, elements from different treatment schools might be worthy additions, for example modes (from ST) and emotion regulation skills (from DBT).

In conclusion, the present study provides a striking example that CPCNs can be predictive of associations between symptom change over time. It thereby suggests that CPCNs are a powerful exploratory tool to generate hypotheses about potentially functional relationship patterns between symptoms and move towards an account of psychopathology that puts these patterns at its core.

## Supporting information

S1 Table Available data by study and time point. (DOCX)

S2 Table Missing data due to dropout by time point for Wetzelaer et al. (2014) trial. (DOCX)

S3 Table Legend for nodes in networks of BPDSI symptom scales. (DOCX)

S1 Fig Partial correlation network of baseline BPDSI symptom scales. See S3 Table for node legend. A minimum parameter of .05 was applied.
(DOCX)

S2 Fig Partial correlation network of random slopes in BPDSI symptom scales. See S3 Table for node legend. Note that nodes were placed in the same location as in S1 Fig for ease of comparison.
(DOCX)

S3 Fig Centrality parameters for partial correlation network of baseline BPDSI symptom scales (see S1 Fig). See S3 Table for node legend.
(DOCX)

S4 Fig Centrality parameters for partial correlation network of baseline BPDSI items (Fig 1, panel A). Items are labeled in the following way: (number of symptom scale. item number).
(DOCX)

S5 Fig Correlations between random slopes of BPDSI subscales and random slopes of the respective BPDSI restscore. See S3 Table for node legend.
(DOCX)

S6 Fig Correlations between random slopes of BPDSI items and random slopes of the respective BPDSI restscore. Items are labeled in the following way: (number of symptom scale. item number).
(DOCX)

S7 Fig Fixed linear time slopes of BPDSI subscales. Slopes were calculated by taking the mean of posterior model distributions and represent change in the subscale score per month. See S3 Table for node legend.
(DOCX)

S8 Fig Fixed linear time slopes of BPDSI items. Slopes were calculated by taking the mean of posterior model distributions and represent change in the item score per month. Items are labeled in the following way: (number of symptom scale. item number).
(DOCX)

S1 Data R code. (DOCX)
