# Supplementary material for: The exploratory value of cross-sectional partial correlation networks: Predicting relationships between change trajectories in borderline personality disorder
Source: PLoS One. 2021 Jul 30;16(7):e0254496. doi: 10.1371/journal.pone.0254496 (PMC8323921; doi:10.1371/journal.pone.0254496)
Supplement: S1 Table — (DOCX) [file pone.0254496.s001.docx]

| S1 Table. Available data by study and time point. | | | | | | | | | | | | | | |
| --- | --- | --- | --- | --- | --- | --- | --- | --- | --- | --- | --- | --- | --- | --- |
| Giesen-Bloo et al. (2006) | | | | | | | | | | | | | | |
|  | months | 0 | 3 | 6 | 9 | 12 | 15 | 18 | 21 | 24 | 27 | 30 | 33 | 36 |
|  | N | 86 | 77 | 73 | 69 | 68 | 64 | 63 | 56 | 57 | 54 | 48 | 47 | 54 |
| Wetzelaer et al. (2014) | | | | | | | | | | | | | | |
|  | months | 0 | 6 | 12 | 18 | 24 | 36 |  |  |  |  |  |  |  |
|  | N | 508 | 377 | 288 | 225 | 202 | 154 |  |  |  |  |  |  |  |
| Dickhaut & Arntz (2014) | | | | | | | | | | | | | | |
|  | months | 0 | 6 | 12 | 18 | 24 | 30 |  |  |  |  |  |  |  |
|  | N | 18 | 14 | 13 | 11 | 11 | 10 |  |  |  |  |  |  |  |
| Leppänen et al. (2015) | | | | | | | | | | | | | | |
|  | months | 0 | 12 |  |  |  |  |  |  |  |  |  |  |  |
|  | N | 71 | 49 |  |  |  |  |  |  |  |  |  |  |  |
