# Supplementary material for: The exploratory value of cross-sectional partial correlation networks: Predicting relationships between change trajectories in borderline personality disorder
Source: PLoS One. 2021 Jul 30;16(7):e0254496. doi: 10.1371/journal.pone.0254496 (PMC8323921; doi:10.1371/journal.pone.0254496)
Supplement: S2 Table — (DOCX) [file pone.0254496.s002.docx]

| S2 Table. Missing data due to dropout by time point for Wetzelaer et al. (2014) trial. | | | | | | | | | | | | | | |
| --- | --- | --- | --- | --- | --- | --- | --- | --- | --- | --- | --- | --- | --- | --- |
|  | months | 0 | 6 | 12 | 18 | 24 | 36 |  |  |  |  |  |  |  |
|  | N | 0 | 66 | 102 | 122 | 125 | 137 |  |  |  |  |  |  |  |
