# Supplementary material for: The exploratory value of cross-sectional partial correlation networks: Predicting relationships between change trajectories in borderline personality disorder
Source: PLoS One. 2021 Jul 30;16(7):e0254496. doi: 10.1371/journal.pone.0254496 (PMC8323921; doi:10.1371/journal.pone.0254496)
Supplement: S3 Table — (DOCX) [file pone.0254496.s003.docx]

| S3 Table. Legend for nodes in networks of BPDSI symptom scales. |
| --- |

| **label** | **BPDSI symptom scale** |
| --- | --- |
| abandon | abandonment |
| relation | interpersonal relationships |
| identity | identity |
| impuls | impulsivity |
| suicid | parasuicidal behavior |
| a. instab | affective instability |
| emptiness | emptiness |
| anger | outbursts of anger |
| disso/para | dissociation or paranoid ideation |
