# Supplementary material for: The exploratory value of cross-sectional partial correlation networks: Predicting relationships between change trajectories in borderline personality disorder
Source: PLoS One. 2021 Jul 30;16(7):e0254496. doi: 10.1371/journal.pone.0254496 (PMC8323921; doi:10.1371/journal.pone.0254496)
Supplement: S2 Fig — See S3 Table for node legend. Note that nodes were placed in the same location as in S1 Fig for ease of comparison. (DOCX) [file pone.0254496.s005.docx]

**S2 Fig*.* Partial correlation network of random slopes in BPDSI symptom scales.** See S3 Table for node legend. Note that nodes were placed in the same location as in S1 Fig for ease of comparison.
