# Supplementary material for: The exploratory value of cross-sectional partial correlation networks: Predicting relationships between change trajectories in borderline personality disorder
Source: PLoS One. 2021 Jul 30;16(7):e0254496. doi: 10.1371/journal.pone.0254496 (PMC8323921; doi:10.1371/journal.pone.0254496)
Supplement: S3 Fig — See S3 Table for node legend. (DOCX) [file pone.0254496.s006.docx]

**S3 Fig*.* Centrality parameters for partial correlation network of baseline BPDSI symptom scales (see S1 Fig).** See S3 Table for node legend.
