# Supplementary material for: The exploratory value of cross-sectional partial correlation networks: Predicting relationships between change trajectories in borderline personality disorder
Source: PLoS One. 2021 Jul 30;16(7):e0254496. doi: 10.1371/journal.pone.0254496 (PMC8323921; doi:10.1371/journal.pone.0254496)
Supplement: S4 Fig — Items are labeled in the following way: (number of symptom scale. item number). (DOCX) [file pone.0254496.s007.docx]

**S4 Fig*.* Centrality parameters for partial correlation network of baseline BPDSI items (Fig 1, panel A)**. Items are labeled in the following way: (number of symptom scale . item number).
