# Supplementary material for: The exploratory value of cross-sectional partial correlation networks: Predicting relationships between change trajectories in borderline personality disorder
Source: PLoS One. 2021 Jul 30;16(7):e0254496. doi: 10.1371/journal.pone.0254496 (PMC8323921; doi:10.1371/journal.pone.0254496)
Supplement: S6 Fig — Items are labeled in the following way: (number of symptom scale. item number). (DOCX) [file pone.0254496.s009.docx]

**S6 Fig. Correlations between random slopes of BPDSI items and random slopes of the respective BPDSI restscore.** Items are labeled in the following way: (number of symptom scale . item number).
