# Supplementary material for: The exploratory value of cross-sectional partial correlation networks: Predicting relationships between change trajectories in borderline personality disorder
Source: PLoS One. 2021 Jul 30;16(7):e0254496. doi: 10.1371/journal.pone.0254496 (PMC8323921; doi:10.1371/journal.pone.0254496)
Supplement: S7 Fig — Slopes were calculated by taking the mean of posterior model distributions and represent change in the subscale score per month. See S3 Table for node legend. (DOCX) [file pone.0254496.s010.docx]

**S7 Fig. Fixed linear time slopes of BPDSI subscales.** Slopes were calculated by taking the mean of posterior model distributions and represent change in the subscale score per month. See S3 Table for node legend.
