# Supplementary material for: The exploratory value of cross-sectional partial correlation networks: Predicting relationships between change trajectories in borderline personality disorder
Source: PLoS One. 2021 Jul 30;16(7):e0254496. doi: 10.1371/journal.pone.0254496 (PMC8323921; doi:10.1371/journal.pone.0254496)
Supplement: S1 Data — (DOCX) [file pone.0254496.s012.docx]

**S1 Data. R-code**

In the following we present code for analyses on BPDSI subscales. This can easily be adapted for BPDSI items.

**Baseline partial correlation network and centrality**

library(huge)

library(qgraph)

library(MCMCglmm)

corMat <- cor(huge.npn(baseline_D[,76:84]), method="pearson")

netW_S <- qgraph(corMat, graph = "glasso", sampleSize = nrow(baseline_D), layout="spring", minimum = .05)

central_S <- centralityPlot(netW_S, standardized = T)

Here baseline_D[,76:84] refers to data in the 9 BPDSI subscales at baseline.

**Estimation of 9 multivariate multi-level linear regression models**

prior1 <- list(R = list(V = .1*diag(2), nu = 3), G = list(G1 = list(V = .1*diag(4), nu = 5), G2 = list(V = .1*diag(4), nu = 5)))

modellist_S <- list()

for (i in 1:9){

X <- D[,c(1,i+75,i+225,73:74)]

colnames(X)[2:3] <- c("symptom", "rest")

modelX <- MCMCglmm(fixed = cbind(symptom, rest) ~ -1 + trait +

trait:time,

random = ~ us(-1 + trait + trait:time):study + us(-1 + trait + trait:time):ID,

rcov = ~ us(trait):units,

family = c("gaussian","gaussian"),

data = X,

nitt = 105000, burnin = 5000, thin = 50,

prior = prior1,

pr = TRUE)

modellist_S[[i]] <- modelX

}

In D[,c(1,76,226,73:74)] the columns respectively refer to the variables ID, BPDSIsubscale1, BPDSIsubscale1_restscore, study, and time.

**Extracting correlations between random slopes**

randomslope_cor <- data.frame(matrix(nrow=2000,ncol=9))

for (i in 1:9){

randomslope_cor[,i] <- modellist_S[[i]]$VCV[,28] / (sqrt(modellist_S[[i]]$VCV[,32]) * sqrt(modellist_S[[i]]$VCV[,27]))

}

Mrandomslope_cor <- colMeans(randomslope_cor)

Note: correlations are extracted by first calculating a posterior distribution from covariance and variance posteriors and then calculating the mean.

**Extracting random slopes and estimating slope network**

RS_D <- data.frame(matrix(nrow=683, ncol=9))

for (i in 1:9){

RS_D[,i] <- colMeans(modellist_S[[i]]$Sol[,1387:2069])

}

corMat_RS <- cor(RS_D, method="pearson")

netWS_RS <- qgraph(corMat_RS, graph="glasso", sampleSize=683, layout=netW_S$layout)
